# Syndromic Surveillance for Local Outbreaks of Lower-Respiratory Infections: Would It Work?

**DOI:** 10.1371/journal.pone.0010406

**Published:** 2010-04-29

**Authors:** Cees C. van den Wijngaard, Liselotte van Asten, Wilfrid van Pelt, Gerda Doornbos, Nico J. D. Nagelkerke, Gé A. Donker, Wim van der Hoek, Marion P. G. Koopmans

**Affiliations:** 1 Centre for Infectious Disease Control, National Institute for Public Health and the Environment, Bilthoven, The Netherlands; 2 United Arab Emirates University, Al-Ain, United Arab Emirates; 3 Netherlands Institute of Health Services Research, Utrecht, The Netherlands; 4 Erasmus Medical Center, Rotterdam, The Netherlands; University of Giessen Lung Center, Germany

## Abstract

**Background:**

Although syndromic surveillance is increasingly used to detect unusual illness, there is a debate whether it is useful for detecting local outbreaks. We evaluated whether syndromic surveillance detects local outbreaks of lower-respiratory infections (LRIs) without swamping true signals by false alarms.

**Methods and Findings:**

Using retrospective hospitalization data, we simulated prospective surveillance for LRI-elevations. Between 1999–2006, a total of 290762 LRIs were included by date of hospitalization and patients place of residence (>80% coverage, 16 million population). Two large outbreaks of Legionnaires disease in the Netherlands were used as positive controls to test whether these outbreaks could have been detected as local LRI elevations. We used a space-time permutation scan statistic to detect LRI clusters. We evaluated how many LRI-clusters were detected in 1999–2006 and assessed likely causes for the cluster-signals by looking for significantly higher proportions of specific hospital discharge diagnoses (e.g. Legionnaires disease) and overlap with regional influenza elevations. We also evaluated whether the number of space-time signals can be reduced by restricting the scan statistic in space or time. In 1999–2006 the scan-statistic detected 35 local LRI clusters, representing on average 5 clusters per year. The known Legionnaires' disease outbreaks in 1999 and 2006 were detected as LRI-clusters, since cluster-signals were generated with an increased proportion of Legionnaires disease patients (p:<0.0001). 21 other clusters coincided with local influenza and/or respiratory syncytial virus activity, and 1 cluster appeared to be a data artifact. For 11 clusters no likely cause was defined, some possibly representing as yet undetected LRI-outbreaks. With restrictions on time and spatial windows the scan statistic still detected the Legionnaires' disease outbreaks, without loss of timeliness and with less signals generated in time (up to 42% decline).

**Conclusions:**

To our knowledge this is the first study that systematically evaluates the performance of space-time syndromic surveillance with nationwide high coverage data over a longer period. The results show that syndromic surveillance can detect local LRI-outbreaks in a timely manner, independent of laboratory-based outbreak detection. Furthermore, since comparatively few new clusters per year were observed that would prompt investigation, syndromic hospital-surveillance could be a valuable tool for detection of local LRI-outbreaks.

## Introduction

The SARS epidemic in 2003, the bioterrorism attacks in 2001, and the ongoing threat of new infectious disease outbreaks have prompted many countries to invest in their capacity to respond timely to emerging infectious disease outbreaks, as early outbreak-detection may well mitigate their impact. As a result, new surveillance systems for earlier detection have been implemented, often labeled “syndromic surveillance” [1]–[6]. These systems use increased reporting of critical symptoms or clinical diagnoses as early indicators of infectious disease outbreaks. This not only allows monitoring of clinical syndromes before laboratory diagnoses have been made, but also allows detection of outbreaks of diseases for which no diagnostics were requested or available (including emerging pathogens). Geographic analysis methods – such as space-time scan statistics – may further increase the sensitivity of syndromic surveillance for detection of local outbreaks or of regional differences in regular seasonal epidemic diseases [2], [6]. In the SARS outbreak in Hongkong in 2003, it is believed that a near real-time space-time analysis would have detected the highly unusual clustering of severe acute respiratory syndrome cases much sooner [7]. However, concerns exist about the specificity of space-time syndromic surveillance, i.e. that it might generate many false signals [8], [9].

The objective of this study was to evaluate to what extent syndromic surveillance detects local outbreaks of lower-respiratory infections (LRIs) without swamping true signals by false alarms. Using retrospective hospitalization data, we simulated prospective space-time syndromic surveillance for LRI-elevations. The two largest outbreaks of Legionnaires' disease in the Netherlands in the last decade were used as “positive controls” to test whether these known outbreaks would have been detected by space-time signals in LRI data. To assess other (likely) causes for detected LRI-elevations, we examined regional increases in the reported incidence of influenza-like-illness (ILI), hospital discharge diagnoses for respiratory illnesses and age group distributions for LRI cases. We also evaluated whether the number of generated space-time signals can be reduced by restricting the time and spatial windows for the analyses.

## Methods

### Ethical Approval

Since we only used anonymous data from existing medical research and surveillance registries, neither formal ethics committee approval nor informed consent from the patients were required.

### LRI-syndrome data (1999–2006)

Hospitalization data were collected from the Dutch National Medical Register (discharge and secondary diagnoses by date of hospitalization for 1999–2006). In 1999–2004 this registry had a 99% coverage (16 million pop.) and in 2005/6 approximately 80%, after exclusion of hospitals with incomplete data for those years.

We included all records on hospitalizations with any kind of LRI as either discharge or secondary diagnosis, under the assumption that this reflects prospective classification of patients with a lower respiratory infection in a “LRI-syndrome” on the day of hospitalization. ICD-9-CM (International Classification of Diseases, 9th revision, Clinical Modification) codes for a LRI syndrome were selected from the CDC respiratory syndrome codes-list (Centers for Disease Control and Prevention, USA, http://www.bt.cdc.gov/surveillance/syndromedef/; and see Appendix S1). After excluding duplicate hospitalizations of the same patient within 6 weeks (5% excluded), 222638 records were included for 1999–2004, and 68124 for 2005–2006. Data were aggregated by hospitalization date, postal-code and age group (0–4, 5–19, 20–49, 50–64, ≥65 years). Since higher levels of spatial resolution can result in more sensitive detection of outbreaks [10], [11] we used 4-digit postal-codes (4023 areas in a 16 million population), which provide the highest level of spatial resolution available within privacy regulations.

### Regional ILI-surveillance data

ILI-data were collected from a sentinel network of general practitioners (GPs, Continuous Morbidity Registration Centres, CMR sentinel stations, 1% population coverage) [12]). The ILI-counts and underlying GP-practice populations were aggregated by region and week. The GP-practice populations were corrected for weeks that specific GP-practices did not supply data. Due to the small number of GP-practices in some parts of the country, the weekly ILI-data were aggregated in 4 major regional groups instead of postal codes.

### Test-case outbreaks

Two large outbreaks of Legionnaires' disease were used as “positive controls” for emerging LRI-outbreaks [13], [14]:

1. In March 1999, a large Legionnaires' disease outbreak occurred among persons who had visited a flower show [13]. Ten patients with pneumonia were admitted to one hospital between March 7^th^ to 11^th^. By March 11^th^, six patients were diagnosed with Legionnaires' disease and an alarm notice was given to hospitals and GPs in the region. Follow-up investigation detected a total of 188 cases, of whom 167 (87%) were hospitalized and 21 (11%) died.
2. Between July 6^th^–28^th^ 2006, 30 Legionnaires' disease cases were identified in Amsterdam, 2 of which were fatal [14]. On July 7^th^ an alarm notice was given. A cooling tower in the town centre was later identified as the outbreak-source.

### Scan statistics for space-time clusters

For the LRI-data, we used a space-time permutation scan statistic which compared the observed number of cases in circular areas with variable radii in flexible time periods vs the expected number of cases, based on the geographic distribution of cases in the whole dataset [15]. In this way, only the case data is needed to estimate the expected number of cases in each space-time window, and population density and time trends in the case data are automatically adjusted for.

We used SaTScan software [16] and the SaTScan Macro Accessory for Cartography (SMAC [17], applied in SAS version 9.1, SAS Institute Inc., Cary, NC, USA) to run the scan-statistic and visualize the results. We simulated a prospective surveillance by running the scan-statistic on data from the year preceding each time unit (day or week) in the analysis period. Thus, weekly or daily space-time signals were generated, each time that the observed number of cases in a certain space and time window exceeded the defined significance threshold. Since such analysis consumes a lot of computation time, we performed weekly analysis (instead of daily) over the whole study period. Daily analyses were also performed in the years that the test-case outbreaks occurred (1999 and 2006), to assess the earliest possible detection date. For all analyses, we chose to use a time-aggregation level of 7-days length. For the daily analyses, these 7-day aggregation windows shifted one day forward for each daily run. Thus we both reduced the computation time and adjusted for day-of-week effects (both purely temporal and spatial day-of-week effects).

To indicate the significance of detected space-time signals, we used recurrence intervals, which indicate how often a signal of the observed significance would be observed by chance under the hypothesis of no outbreak [18]. I.e. if the recurrence interval of a signal is say 1 year, 1 signal of the observed significance is expected in 1 year. Two thresholds levels were used: signals with recurrence interval ≥1 and ≥5 years. We assessed whether successive signals overlapped in space and time, which suggests the same cause. For the sake of readability, we indicated a group of such overlapping space-time signals as “cluster” and an individual space-time signal as “cluster-signal”.

**Figure 1:**
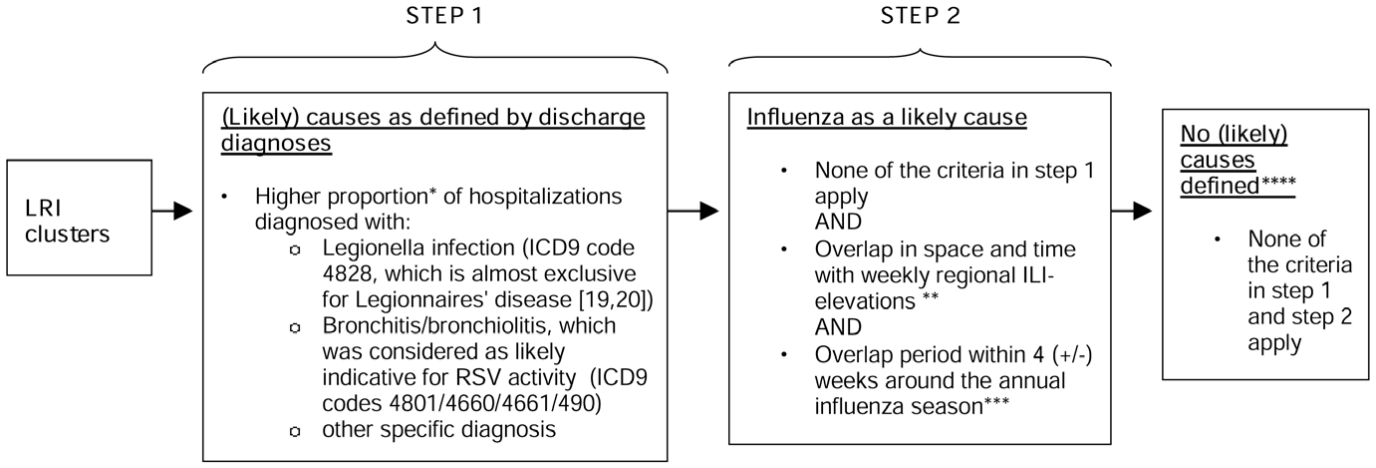
Two-step criteria to define (likely) causes for LRI hospitalization clusters detected in 1999–2006. * As evaluated by the right-sided Fisher's exact test for 2×2 Tables (alpha≤0.01) of hospitalizations within vs hospitalizations outside of the cluster-signal. The proportion of hospitalizations with a specific characteristic (e.g. legionnaires' disease as discharge diagnoses, or age 20–49 yrs) can be significantly higher among hospitalizations within the cluster-signal than the proportion outside of the cluster-signal. ** For the ILI-cluster-signals we could only use 4 major regions as spatial resolution. Overlap in time between LRI and ILI-cluster-signals was defined as occurrence of weekly ILI-cluster-signals within 2 weeks (+/−) around LRI-cluster-signals. ***The annual influenza season was defined as all weeks with a national weekly ILI-incidence ≥3 per 10.000 pop. **** Possibly unreported/undetected local LRI-outbreaks by undetected pathogens.

We evaluated how many LRI-clusters and signals were detected over the whole study period (1999–2006) and looked for explanations guided by the two-step criteria in Figure 1. In step one, we assessed likely causes for the cluster-signals by looking for significantly higher proportions of specific hospital discharge diagnoses (e.g., Legionnaires' disease [19], [20]). In step two we assessed overlap with regional ILI clusters (Appendix S2), as (local) influenza activity might be reflected in local LRI-elevations. Since other pathogens than influenza might cause some ILI fluctuations, influenza activity was only considered to be a likely cause if space-time overlap between LRI and ILI-clusters coincided with the annual influenza season (Figure 1). If a specific cause was defined for one or more signals within one cluster, we considered that to be a likely cause for the whole cluster. We also evaluated the timeliness of detection for the clusters related to the known Legionnaires' disease outbreaks.

A sensitivity analysis was used to evaluate the impact of time and spatial window settings on the number of clusters and signals detected. For the initial analyses, we put only minor constraints on the maximum temporal and spatial windows of the scan-statistic, to avoid wrongful assumptions about time, geographical location and size of an outbreak. We then repeated these weekly analyses with a temporal window of maximum 7 weeks and also with a spatial window of maximum 25 km radius, to assess the impact of these parameters on the number of signals generated.

See Appendix S2 for further details on use and settings of the scan-statistics.

## Results

### LRI-clusters

**Figure 2:**
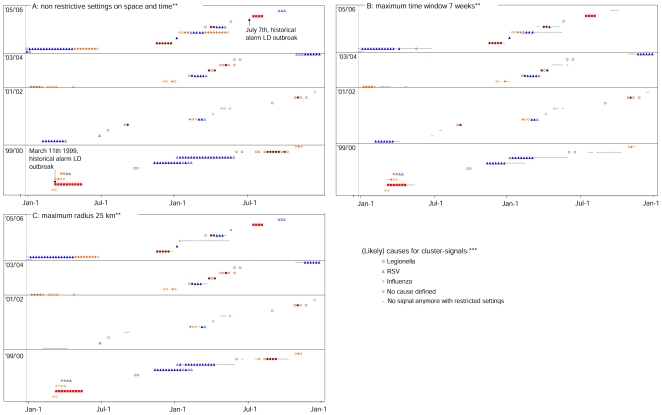
Clusters and generated cluster-signals on a timescale, including all (likely) causes (by weekly analysis).* *Clusters are indicated by sets of successive space-time overlapping cluster-signals placed next to each other on the same height on the y-axis. The cluster-signals caused by a data artifact in 2000 are not presented in the graphs. See Figure 1 for the criteria by which the likely causes were defined and see the Figure 2 legend for the graphic indication of likely causes. **In Figure 2a — for the analyses with non-restrictive settings on time and spatial windows — all detected clusters and signals are presented, as well as the (likely) causes according to the criteria in Figure 1. Figure 2b presents the signals and clusters that are still detected with a maximum time window of 7 weeks, and Figure 2c signals and clusters still detected with a maximum radius of 25 km. ***Signals indicated by open symbols (e.g. “○”) have a ≥1 year recurrence interval, coloured symbols (e.g. “•”) have a ≥5 yr recurrence interval. A recurrence interval reflects how often a signal of the observed significance level would be observed by chance [18]. I.e. if the recurrence interval of a signal is say 1 year, 1 signal of the observed significance is expected in 1 year.

Between Feb 1^st^ 1999 and Sept 30^th^ 2006, a total of 35 LRI-clusters with 221 cluster-signals were detected by weekly analysis (Table 1, non-restrictive parameter settings, recurrence interval ≥1 year). By raising the threshold (recurrence interval ≥5 years), we observed only 24 clusters with 146 cluster-signals (respectively 31% and 34% decrease). Figure 2a shows all LRI-clusters and signals on a timescale for the different recurrence interval levels – as detected with the initial non-restrictive parameter settings for space and time windows. The time between the first and the last signal within one cluster ranged from 0 to 26 weeks. By daily analysis, in 1999 and 2006 a total of 194 cluster-signals were detected (compared to 75 signals by weekly analysis with a ≥1 year recurrence level, both with non-restrictive parameter settings). However, the number of clusters was lower (10 clusters by daily analysis vs 12 by weekly analysis in 1999 and 2006).

**Table 1:** Detected LRI-clusters and signals between 1999 Feb 1^st^ and 2006 Sept 30^th^ by weekly analysis (recurrence interval ≥1 or ≥5 years) for different parameter settings.

	(A) Non restrictive settings for time and spatial windows				(B) Maximum 7 weeks time window				(C) Maximum radius 25 km			
(Likely) cause	LRI-cluster-signals		LRI-clusters*		LRI-cluster-signals		LRI-clusters*		LRI-cluster-signals		LRI-clusters*	
	Recurrence interval		Recurrence interval		Recurrence interval		Recurrence interval		Recurrence interval		Recurrence interval	
	≥1 yr.	≥5 yr.	≥1 yr.	≥5 yr.	≥1 yr.	≥5 yr.	≥1 yr.	≥5 yr.	≥1 yr.	≥5 yr.	≥1 yr.	≥5 yr.
Legionnaires' disease outbreak 1999	10	10	1	1	7	7	1	1	10	10	1	1
Legionnaires' disease outbreak 2006	4	4	1	1	4	4	1	1	4	4	1	1
Local RSV activity	99	78	9	7	62	55	7	7	68	56	8	6
Local influenza activity	55	28	8	5	25	13	7	5	40	17	9	4
Local RSV *and* influenza activity	n/a	n/a	4	3	n/a	n/a	4	3	n/a	n/a	3	2
Other specific pathogen**	7	6	1	1	5	5	1	1	7	6	1	1
No cause defined***	46	20	11	6	26	12	9	4	36	16	10	5
**Total**	221	146	35	24	129	96	30	22	165	109	33	20

The total number of detected clusters and signals is presented, for the non-restrictive parameter settings on space and time (A), for the settings with a maximum time window of 7 weeks (B), and for the settings with a maximum radius of 25 km (C). The distribution of (likely) causes according to the criteria in Figure 1 is also presented in the Table.

* A cluster is defined by a set of successive cluster-signals that overlap in space and time.

** The cluster-signals in this category formed only one cluster, which appeared to be caused by a data artifact.

*** Possibly unreported/undetected local LRI-outbreaks by undetected pathogens.

**Figure 3:**
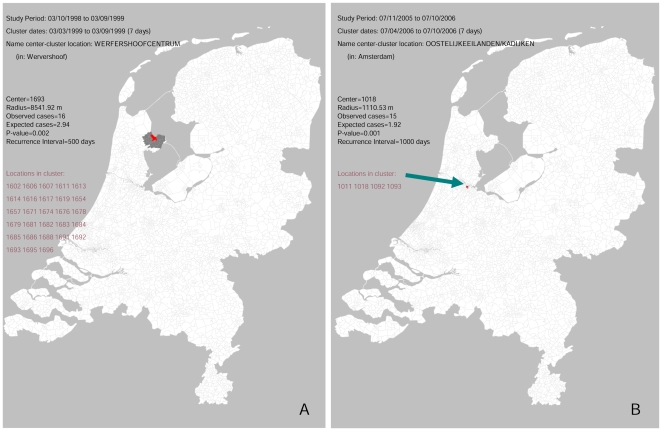
The earliest detected Legionnaires' disease outbreak related LRI-cluster-signals (1999 and 2006) as presented on a map of the Netherlands (by daily analysis). Figure 3a and 3b show the cluster-signals that detected the 1999 and 2006 outbreak respectively. Output of the Satscan scan-statistic software is presented in the legend. On the map the borders of all postal code areas are indicated, the postal code areas of the cluster-signals are marked in dark-grey with the center postal code marked in red.

Figure 2a and Table 1 also show the likely causes for the detected LRI-clusters (according to the criteria in Figure 1, see methods section). The known Legionnaires' disease outbreaks in 1999 and 2006 were detected by LRI-clusters, since cluster-signals were generated with an increased proportion of patient discharge diagnoses for Legionnaires' disease in both outbreak areas and periods (Table 1, Figure 2a and 3a–b) (proportions differed between successive signals: 44–65% in 1999, and 21–63% in 2006; p:<0.0001). The 1999 Legionnaires' disease related cluster-signals included a higher proportion of persons 50–64 years of age (37–48%; p:<0.0001). We compared the earliest detection dates for these outbreaks for daily and weekly analysis. Daily analysis signaled the outbreak 4 days earlier than weekly analysis, 2 days before the national alarm was given during the 1999 Legionnaires' disease outbreak. The 2006 Legionnaires' disease outbreak was detected by weekly analysis on 2006 July 15^th^, and could have been detected by daily analysis 5 days earlier, 3 days after the national alarm was given.

Many of the other clusters and signals seemed to be related to local RSV and/or influenza activity (70% of cluster-signals and 60% of clusters, Table 1). Some of the influenza and RSV related clusters tended to persist over longer periods (Figure 2a). Young children (0–4 years old) were overrepresented in 82 of the 99 cluster-signals that we scored as RSV related (Table 1; p:<0.05).

In 2000, a cluster was detected with an unusually high number of patients diagnosed with aspergillosis, which was traced to a registration error (one patient was accidentally registered under 28 different anonymous identifiers).

For 46 cluster-signals we did not find a “likely cause” according to the criteria in Figure 1. Of these, 6 belonged to influenza and/or RSV related clusters (Figure 2a), and 11 coincided with local ILI-elevations outside the influenza season (1 at the end of spring 2000, 4 at the end of summer 2000 and 6 at the end of 2005).

When repeating the weekly analyses with restricted time or spatial windows, both Legionnaires' disease outbreaks were still detected with the same timeliness. Table 1 and Figure 2b and 2c also show the clusters and signals that were still detected with a temporal window of maximum 7 weeks, and with a spatial window of maximum 25 km respectively (as compared to the signals detected with the initial non-restrictive settings).

With a time window of maximum 7 weeks, 129 of the 221 initial cluster-signals and 30 of the initial 35 clusters were still detected (respectively 42% and 14% decline, Table 1). Of the 5 clusters not detected — as compared to the initial analyses — 2 had been scored as likely due to RSV, 1 to influenza and for the other 2 no likely cause had been scored (Table 1 and Figure 2a–b).

With a maximum 25 km radius, 165 of the 221 initial cluster-signals and 33 of the 35 clusters were still detected (respectively 25% and 6% decline, Table 1). One of the 2 undetected clusters had been scored as likely due to RSV, for the other no likely cause had been scored (Table 1, Figure 2a and 2c).

Some of the cluster-signals detected with restrictive time/spatial windows had not been detected with the initially detected signals (data not shown). With the restrictive time window 2 borderline significant cluster-signals were detected, that had been non-significant in the initial analysis. This was due to the fact that the restrictive settings limited the adjustments for taking into account the multiple testing (stemming from the many potential cluster locations and sizes evaluated) [15]. With the restrictive spatial window 3 extra cluster-signals were detected due to the same mechanism, and 2 other extra cluster-signals were detected due to the fact that initial cluster-signals that geographically overlapped with them had dropped out.

## Discussion

In this study, prospective surveillance of hospitalization data was simulated using retrospective data, to evaluate whether syndromic surveillance can effectively detect local outbreaks of lower-respiratory infections (LRIs). Over 1999–2006 (400 weeks), 35 space-time LRI-clusters were detected by weekly analysis, with a total of 221 generated cluster-signals. This represents an average rate of approximately 5 new clusters per year, or 3 per year using a threshold recurrence interval ≥5 years. The number of clusters detected per year differed over the study period, reflecting substantial annual variation in influenza epidemics.

Two clusters were related to the Legionnaires' disease “test-case” outbreaks and would have been detected around the same time as the outbreaks were actually detected. This indicates that syndromic surveillance will pick up similar outbreaks of severe respiratory disease in a timely manner. Note that the Legionnaires' disease outbreaks are used here as “positive controls” (or Gold Standard) for realistic severe respiratory outbreaks by uncommon pathogens that may not be (timely) detected by traditional surveillance, such as the Dutch Q-fever outbreak in 2007, for which the initial diagnoses were delayed by several weeks [21], [22]. As 17 out of the total 35 LRI clusters probably reflected local RSV and/or influenza activity, many signal investigations could be limited to checking their concurrence with local RSV and/or influenza activity. The 3 clusters with “unknown cause”, that concur with local ILI-elevations outside the influenza season, possibly represent very early local influenza activity or local activity of another respiratory pathogen reflected in both GP-ILI-data and hospital LRI-data. For these 3 clusters and the other 8 clusters for which no likely cause was defined, it would have been interesting to investigate possible causes in a truly prospective setting (e.g., by additional diagnostics). Some of these clusters possibly represent unreported and/or undetected local LRI-outbreaks.

As a threshold value for the significance of cluster signals, we used a threshold of recurrence intervals ≥1 year, and only evaluated the LRI clusters that were above this threshold. To illustrate the impact of changing the threshold we repeated the analyses for recurrence intervals ≥5 years. At both threshold levels, two LRI clusters showed a higher proportion of Legionnaires' disease cases (p:<0.0001, see also results section) overlapping with the known outbreak areas, which made us conclude that these LRI clusters indeed detected the Legionnaires' disease outbreaks.

The results of the sensitivity analysis show that the test outbreaks are still detected with the restricted time and spatial windows (at both threshold levels), without loss of timeliness and with less signals generated in time. To limit the computation time we only performed a modest sensitivity analysis. In this study, the restrictions on the time window almost halved the number of signals (42% decline), whereas the clusters in time to investigate declined much less (14% decline). The spatial restrictions resulted in less decline in generated signals (25% decline in signals and 6% decline in clusters). This indicates that with little loss of sensitivity, the restricted time window would be most appropriate to limit the number of generated signals.

To our knowledge this is the first study that evaluates the performance of syndromic surveillance with nationwide high coverage data (80–99% of hospitalizations) over a longer period (8 years) with all detected clusters analyzed and (if possible) explained in a systematic way. Feasibility of localized outbreak detection is demonstrated without swamping true signals by excessive false alarms. Some other studies evaluating the performance of space-time syndromic surveillance have concluded differently, but these studies were based on shorter periods, had lower coverage or lacked comparable outbreaks which could be tested [8], [23], [24]. Cooper et al. tracked the spatial diffusion of influenza and norovirus, using space-time analysis on syndromic data from a telephone help line system in the UK, but did not test space-time detection for more localized outbreaks [23]. Using syndromic surveillance for detection of local gastro-intestinal outbreaks in New York City, Balter et al. found numerous cluster-signals in time, but these could not be used for effective surveillance because of insufficient comparable diagnostic data [8]. Respiratory disease outbreaks could not be evaluated in the NYC study, because no local respiratory outbreaks had been reported in the study period. Nordin et al. used simulated anthrax attack data injected in true physician's visit data to confirm that a respiratory outbreak initiated by bioterrorism will be detected in a timely manner by syndromic surveillance [24]. However, no results on the number of possibly false alarms were presented. These studies present space-time cluster detection analyses over relatively few years and are therefore prone to miss the effects of annual variation. Furthermore, sensitivity for local outbreaks is reduced by using data with relatively low coverage levels. For such data sources with low coverage, methods other than space-time scan statistics seem more appropriate to generate useful information for public health practice (like aberration detection in time).

We performed weekly analyses (instead of daily) over the whole study period, because these analyses consume considerable computation time. Daily analyses in 1999 and 2006 detected fewer clusters than weekly analyses because the threshold level for recurrence intervals (≥1 year) is more strict (see Appendix S2). Daily analyses would therefore probably not detect more epidemiological events but would yield more timely signals.

Hospital based syndromic surveillance could be a helpful tool in detecting local LRI-outbreaks, complementing outbreak detection by laboratory surveillance or astute clinicians. Syndromic surveillance might be most valuable for outbreaks due to uncommon or novel pathogens (like the SARS outbreak), as these seem more likely to be missed by the laboratory and clinicians. Furthermore, outbreaks due to more common pathogens could also be missed, as for community acquired pneumonia often no causative pathogen is detected [25], [26]. Apart from that, under-notification can complicate outbreak detection through laboratories and clinicians [20].

A prerequisite for prospective syndrome surveillance is the real-time availability of hospitalization data, including clinical diagnoses and symptoms by date of hospitalization. Although at present not available in the Netherlands, such real-time syndromic data collection may become feasible after the nationwide implementation of electronic health-care information exchange. In this light, the results of our study justify further development of these methods, including retrospective evaluation of other types of documented health events than the ones presented in our study.

Besides that, further research should focus on prospective application of these methods. In a prospective setting, sustaining reliable data with high coverage and few data artifacts might be more challenging, thus possibly leading to higher numbers of false alarms. In addition, it should be evaluated to what extent 3 to 5 new syndromic clusters per year would indeed be manageable in a prospective setting. Responding to such clusters is complicated, because the cause and thus possible threat will initially often be unknown. For each new cluster, it should first be verified whether plausible explanations can be found in epidemiological or laboratory data. For example, LRI clusters need to be interpreted in relation to local influenza or RSV activity similar as we did in our study, and provided the age distribution of cases reflects the usual pattern, further investigation would seem unnecessary. Internet-based ILI-monitoring [27] combined with virological self -sampling (at home) [28] could increase the microbiological base for interpreting syndromic surveillance data. Age stratified syndromic surveillance with a multivariate space-time scan statistic [29] may further facilitate quick interpretation of clusters by revealing the affected age groups.

### Conclusion

This retrospective study shows that space-time syndromic surveillance on hospitalizations can timely detect local LRI-outbreaks independent of detection of the causative pathogen. The frequency of cluster detection, when interpreted in the light of available epidemiological and microbiological data, does not give rise to excessive levels of further investigations.

Consequently, we recommend real-time syndromic surveillance as an additional tool for detection of local LRI outbreaks, but only if syndromic data with sufficient quality and coverage can be collected, coupled with epidemiological and microbiological data. Public health responses can be based on a combination of syndromic surveillance data, reports by astute clinicians and early diagnostic test results, which all could generate the first alarm for different kinds of disease events. Future research on prospective syndromic surveillance should therefore focus on practical methods for integrating syndromic surveillance alarms with clinical reports and laboratory information for effective public-health responses.

## Supporting Information

Appendix S1 Detailed syndrome definition for hospitalizations with lower-respiratory infection syndrome.
(0.06 MB DOC)

Appendix S2 Details on space-time analyses and Satscan settings.
(0.03 MB DOC)
