# Supplementary material for: Syndromic Surveillance for Local Outbreaks of Lower-Respiratory Infections: Would It Work?
Source: PLoS One. 2010 Apr 29;5(4):e10406. doi: 10.1371/journal.pone.0010406 (PMC2861591; doi:10.1371/journal.pone.0010406)
Supplement: Appendix S1 — Detailed syndrome definition for hospitalizations with lower-respiratory infection syndrome. (0.06 MB DOC) [file pone.0010406.s001.doc]

**Appendix S1: Detailed syndrome definition for hospitalizations with lower-respiratory infection syndrome.**

We used discharge and secondary diagnoses on date of hospitalization from the Dutch National Medical Register (LMR, 99% coverage over 1999-2004, 80 % coverage over 2005-2006 16 million pop., coded in Dutch version of ICD-9-CM).

To define a lower-respiratory infection syndrome, we selected ICD-9-CM (International Classification of Diseases, 9th revision, Clinical Modification) codes for any kind of lower-respiratory infection from the respiratory syndrome codes-list as selected by the CDC (Centers for Disease Control and Prevention, USA, <http://www.bt.cdc.gov/surveillance/syndromedef/>). See the table below for all selected lower-respiratory infection codes. We selected these codes as a subset from the set of codes for general respiratory symptoms and diagnoses (‘category 1’ in CDC-list) and the codes for specific respiratory biologic agent diagnoses (‘category 3’ in CDC-list). Finally these syndrome codes were slighty adapted for the Dutch version of ICD-9-CM.

Table. ICD9-CM codes for lower-respiratory infection syndrome in hospitalization data.

| **ICD9-CM** | **Description** |  |  |  |  |
| --- | --- | --- | --- | --- | --- |
| 003.22 | SALMONELLA PNEUMONIA | | | | |
| 020.3 | PRIMARY PNEUMONIC PLAGUE | | | | |
| 020.4 | SECONDARY PNEUMON PLAGUE | | | | |
| 020.5 | PNEUMONIC PLAGUE NOS | | | | |
| 021.2 | PULMONARY TULAREMIA | | | | |
| 022.1 | PULMONARY ANTHRAX | | | | |
| 031.0 | MYCOBACTERIA, PULMONARY | | | | |
| 052.1 | VARICELLA WITH PNEUMONIA | | | | |
| 055.1 | POSTMEASLES PNEUMONIA | | | | |
| 073.0 | ORNITHOSIS, WITH PNEUMONIA | | | | |
| 114.0 | PRIMARY COCCIDIOIDOMYCOSIS (LUNG) | | | |  |
| 114.9 | COCCIDIOIDOMYCOSIS NOS | | | | |
| 115.05 | *HISTOPLASMA CAPSULATUM* PNEUMONIA | | | | |
| 115.15 | *HISTOPLASMA DUBOISII* PNEUMONIA | | | | |
| 115.95 | HISTOPLASMOSIS PNEUMONIA | | | | |
| 116.0 | BLASTOMYCOSIS | | | | |
| 116.1 | PARACOCCIDIOIDOMYCOSIS | | | | |
| 117.3 | PULMONARY ASPERGILLOSIS | | | | |
| 130.4 | TOXOPLASMA PNEUMONITIS | | | | |
| 136.3 | PNEUMOCYSTOSIS | | | | |
| 466.0 | BRONCHITIS ACUTE | | | | |
| 466.1 | ACUTE BRONCHIOLITIS | |  |  |  |
| 480.0 | ADENOVIRAL PNEUMONIA | | | | |
| 480.1 | PNEUMONIA DUE TO RESPIRATORY SYNCYTIAL VIRUS | | | | |
| 480.2 | PARINFLUENZA VIRAL PNEUMONIA | | | | |
| 480.8 | VIRAL PNEUMONIA NEC | | | | |
| 480.9 | PNEUMONIA, VIRAL | | | | |
| 481 | PNEUMOCOCCAL PNEUMONIA (LOBAR) | | | | |
| 482.0 | PNEUMONIA DUE TO KLEBSIELLA PNEUMONIAE | | | | |
| 482.1 | PNEUMONIA DUE TO PSEUDOMONAS | | | | |
| 482.2 | *HAEMOPHILUS INFLUENZAE* PNEUMONIA | | | | |
| 482.3 | PNEUMONIA DUE TO STREPTOCOCCUS | | | |  |
| 482.4 | PNEUMONIA DUE TO STAPHYLOCOCCUS | | | |  |
| 482.8 | PNEUMONIA DUE TO BACT. NEC | | |  |  |
| 482.9 | PNEUMONIA, BACTERIAL NOS | | | | |
| 483 | PNEUMONIA DUE TO ORGANISM NEC | | | |  |
| 484.1 | PNEUMONIA DUE TO CYTOMEGALIC INCLUSION DISEASE | | | | |
| 484.3 | PNEUMONIA IN WHOOPING COUGH | | | | |
| 484.5 | PNEUMONIA IN ANTHRAX | | | | |
| 484.6 | PNEUMONIA IN ASPERGILLOSIS | | | | |
| 484.7 | PNEUMONIA IN OTHER SYSTEMIC MYCOSES | | | | |
| 484.8 | PNEUMONIA IN INFECTIOUS DISEASE NEC | | | | |
| 485 | BRONCHOPNEUMONIA ORGANISM UNSPEC | | | | |
| 486 | PNEUMONIA, ORGANISM NOS | | | | |
| 487.0 | INFLUENZA WITH PNEUMONIA | | | | |
| 490 | BRONCHITIS NOS | | | | |
| 511.1 | PLEURISY WITH EFFUSION, WITH MENTION OF A BACTERIAL CAUSE OTHER THAN TUBERCULOSIS | | | | |
