# Supplementary material for: Syndromic Surveillance for Local Outbreaks of Lower-Respiratory Infections: Would It Work?
Source: PLoS One. 2010 Apr 29;5(4):e10406. doi: 10.1371/journal.pone.0010406 (PMC2861591; doi:10.1371/journal.pone.0010406)
Supplement: Appendix S2 — Details on space-time analyses and Satscan settings. (0.03 MB DOC) [file pone.0010406.s002.doc]

**Appendix S2: Details on space-time analyses and Satscan settings**

For detection of space-time clusters in the LRI-hospitalizations data, we used a space-time permutation scan statistic that compares observed and expected numbers of cases in circular areas with variable radii in flexible time periods. A likelihood ratio is calculated for each space-time window, to indicate to what extent the rate of cases inside the area is higher than expected. Monte Carlo hypothesis testing is then used to indicate the significance level of specific space-time windows. As expected numbers are calculated from the geographic distribution of cases in the whole dataset this method does not require additional population-at-risk data, and population density and seasonal variation in the case data is automatically adjusted for [15].

We simulated a prospective surveillance by running the scan-statistic on data from the year preceding each time unit (day or week) in the analysis period. This way, weekly or daily space-time signals were generated. For analyses in 1999, we used data from 2000 as historical data (by generating a stand-in-dataset for 1998 based on data from 2000). For all analyses, we chose to use time aggregation windows of 7-days length, even for the daily analysis for 1999 and 2006. For these daily analyses, the 7-day windows shifted one day forward for each daily run. Thus we both limited the computation time and adjusted for day-of-week effects (both purely temporal and spatial day-of-week effects). To further limit the computation time - for the initial analyses - we chose a maximum spatial cluster-signal size of 40% population at risk (instead of default 50%, the cases are here the population) and a maximum temporal cluster-signal size of 21 weeks (to not only detect outbreaks that evolve in e.g. 1 or 2 weeks, but also more gradually evolving outbreaks).

As a covariate we included the age group (0-4, 5-19, 20-49, 50-64, >=65 years).

To measure the significance of the detected cluster-signals we used recurrence-intervals. The recurrence interval reflects how often a signal of the observed significance level would be observed by chance, assuming that analyses are repeated on a regular basis (e.g. daily/weekly) [18]. E.g. a signal with a recurrence interval of 1 year would, on average, be observed every year. The recurrence interval can be extracted directly from the p-value: e.g. for daily analysis the recurrence interval in days can be calculated as “1/p-value” whereas for weekly analysis the recurrence interval in days is “7/p-value”. This implies that a recurrence interval of 1 year corresponds with a p-value of 0.00274 for daily analyses and 0.0192 for weekly analyses.

A sensitivity analysis was used to evaluate the impact of time and spatial window settings on the number of space-time signals detected. For the initial analyses, as described above we put only minor constraints on the maximum temporal and spatial windows of the scan-statistic. We then repeated these weekly analyses with a temporal window of maximum 7 weeks and also with a spatial window of maximum 25 km radius (arbitrarily chosen). We then used the initial analyses results as reference for the analyses with restricted settings, and evaluated what signals and clusters from the initial analyses were still detected with the restricted settings (by assessing geographical overlap between signals generated at the same date). Restrictions on space and time will lead to (little) extra signals as well, as borderline significant signals as detected with restricted settings, might have been not significant with non restrictive settings - due to more adjustments for taking into account multiple testing - stemming from the many (more) potential cluster locations and sizes evaluated [15].

We also performed space-time scans on the ILI-data, to assess whether regional ILI-clusters might explain detected LRI-clusters. For the ILI-data prospective space-time scans were performed, with a Poisson-distributed number of events in a geographical area, according to a known underlying population-at-risk [30]. Here we chose to use population-at-risk data, because of variation in the number of GP-practices supplying data (e.g. during vacations). We adjusted for purely temporal clusters using the time-stratified randomization option of the Satscan software, to prevent concurring ILI-clusters in all regions, e.g. by seasonal variation. In contrast with the LRI-analyses we only used recurrence intervals of >= 1 year, we divided the Netherlands in 4 major regions as spatial input, we used an analysis period of 180 days back from each date the prospective scan was performed for, we took a maximum spatial cluster-signal size of 50% population at risk (default) and we used a maximum temporal cluster-signal size of 7 days (to only detect weekly cluster-signals corresponding with the weekly regional ILI-incidence fluctuations as reported in the Dutch Influenza News letter, http://www.virology.nl/files/new.pdf). No covariate (age) was used and spatially the clusters were limited to contain only 1 out of the 4 major regions.
